# Adapting Rapid Diagnostic Tests to Detect Historical Dengue Virus Infections

**DOI:** 10.3389/fimmu.2021.703887

**Published:** 2021-07-23

**Authors:** Fernando Echegaray, Peter Laing, Samantha Hernandez, Sully Marquez, Amanda Harris, Ian Laing, Adam Chambers, Neil McLennan, Victor A. Sugiharto, Hua-Wei Chen, Sandra Vivero Villagran, Abigail Collingwood, Magelda Montoya, Fausto Bustos Carrillo, Mark P. Simons, Philip J. Cooper, Andrea Lopez, Gabriel Trueba, Joseph Eisenberg, Shuenn-Jue Wu, William Messer, Eva Harris, Josefina Coloma, Leah C. Katzelnick

**Affiliations:** ^1^ Viral Epidemiology and Immunity Unit, Laboratory of Infectious Diseases, National Institute of Allergy and Infectious Diseases, National Institutes of Health, Bethesda, MD, United States; ^2^ Excivion Ltd., Cambridge, United Kingdom; ^3^ Division of Infectious Diseases and Vaccinology, School of Public Health, University of California, Berkeley, Berkeley, CA, United States; ^4^ Instituto de Microbiología, Universidad San Francisco de Quito, Quito, Ecuador; ^5^ Oxford Expression Technologies Ltd., Oxford, United Kingdom; ^6^ GlobalDX Ltd., Mensrie, United Kingdom; ^7^ Viral & Rickettsial Diseases Department, Naval Medical Research Center, Silver Spring, MD, United States; ^8^ Instituto de Investigación en Biomedicina (Inbiomed), Universidad Central, Quito, Ecuador; ^9^ Department of Epidemiology, University of Michigan School of Public Health, Ann Arbor, MI, United States; ^10^ Department of Infection and Immunity, St George’s University of London, London, United Kingdom; ^11^ School of Medicine, Universidad International del Ecuador, Quito, Ecuador; ^12^ Department of Molecular Microbiology and Immunology, Oregon Health and Sciences University, Portland, OR, United States

**Keywords:** dengue, rapid diagnostic test, pre-vaccination screening, Zika, dengue vaccine

## Abstract

The only licensed dengue vaccine, Dengvaxia^®^, increases risk of severe dengue when given to individuals without prior dengue virus (DENV) infection but is protective against future disease in those with prior DENV immunity. The World Health Organization has recommended using rapid diagnostic tests (RDT) to determine history of prior DENV infection and suitability for vaccination. Dengue experts recommend that these assays be highly specific (≥98%) to avoid erroneously vaccinating individuals without prior DENV infection, as well as be sensitive enough (≥95%) to detect individuals with a single prior DENV infection. We evaluated one existing and two newly developed anti-flavivirus RDTs using samples collected >6 months post-infection from individuals in non-endemic and DENV and ZIKV endemic areas. We first evaluated the IgG component of the SD BIOLINE Dengue IgG/IgM RDT, which was developed to assist in confirming acute/recent DENV infections (n=93 samples). When evaluated following the manufacturer’s instructions, the SD BIOLINE Dengue RDT had 100% specificity for both non-endemic and endemic samples but low sensitivity for detecting DENV seropositivity (0% non-endemic, 41% endemic). Sensitivity increased (53% non-endemic, 98% endemic) when tests were allowed to run beyond manufacturer recommendations (0.5 up to 3 hours), but specificity decreased in endemic samples (36%). When tests were evaluated using a quantitative reader, optimal specificity could be achieved (≥98%) while still retaining sensitivity at earlier timepoints in non-endemic (44-88%) and endemic samples (31-55%). We next evaluated novel dengue and Zika RDTs developed by Excivion to detect prior DENV or ZIKV infections and reduce cross-flavivirus reactivity (n=207 samples). When evaluated visually, the Excivion Dengue RDT had sensitivity and specificity values of 79%, but when evaluated with a quantitative reader, optimal specificity could be achieved (≥98%) while still maintaining moderate sensitivity (48-75%). The Excivion Zika RDT had high specificity (>98%) and sensitivity (>93%) when evaluated quantitatively, suggesting it may be used alongside dengue RDTs to minimize misclassification due to cross-reactivity. Our findings demonstrate the potential of RDTs to be used for dengue pre-vaccination screening to reduce vaccine-induced priming for severe dengue and show how assay design adaptations as well quantitative evaluation can further improve RDTs for this purpose.

## Introduction

Dengue viruses 1 to 4 (DENV1-4) are mosquito-borne flaviviruses that cause about 105 million infections globally each year (1). Approximately 25% of DENV infections cause symptomatic febrile episodes and ~500,000 result in hospitalizations annually (1–4). DENV1-4 are considered serotypes (5). Primary infection with one DENV serotype induces long-term immunological protection against that specific serotype but can increase the risk of developing severe dengue disease during secondary infection by one of the other three serotypes (6, 7). However, after recovering from a secondary DENV infection, individuals are much less likely to develop severe dengue disease upon subsequent infection (8). This has led researchers to conclude that having sequential DENV infections with two distinct serotypes can induce enduring immunological cross-protection against all four DENV serotypes (9).

The only licensed dengue vaccine, Dengvaxia^®^ (Sanofi Pasteur, Lyon, France), also was shown to provide protection against future disease in children vaccinated after they have had at least one prior DENV infection (10). However, Dengvaxia^®^ increased the risk of severe breakthrough DENV infections in children vaccinated when seronegative (10). For this reason, in 2018 the Strategic Advisory Group of Experts on Immunization for the World Health Organization (WHO) recommended using serological assays, such as rapid diagnostic tests (RDT), to provide evidence of prior DENV infection and suitability for vaccination (11). Individuals identified as DENV-seropositive by such assays would be eligible for vaccination while those identified as DENV-seronegative would not. In response, in 2019 the Global Dengue and Aedes-transmitted diseases Consortium (GDAC) recommended that assays have optimal specificity (≥98%) and sensitivity (≥95%) when used for this purpose, with a minimum specificity and sensitivity of 90% (12).

When testing for DENV serostatus, “specificity” refers to the ability of a test to accurately identify those who are truly DENV-naïve as negative. In the context of dengue pre-vaccination screening, high specificity is required to avoid vaccinating individuals who are truly naive and putting them at risk (12, 13). Conversely, “sensitivity” refers to the ability of that test to accurately identify those who have had a prior DENV infection as positive. High sensitivity in dengue pre-vaccination screening assays maximizes detection of individuals with a single prior DENV infection who would benefit from vaccination because they are at greater risk for developing severe dengue disease (6). In 2019, Sanofi Pasteur, the developer of Dengvaxia^®^, evaluated the specificity and sensitivity of four dengue RDTs (Bio-Rad RDT Dengue IgA/IgG, CTK Biotech OnSite Dengue IgG/IgM, SD BIOLINE Dengue IgM/IgG, and GenBody Dengue IgG/IgM) and observed high specificity overall (>98%) but lower sensitivities (40-70%) (14). Further, all four dengue RDTs failed to detect antibodies in individuals with DENV neutralizing antibody titers of <1:100, the group at greatest risk for future severe dengue (6). Sensitivity was also low (0-32%) for sera from individuals with DENV neutralizing antibody titers of 1:100-1:500 (14).

Moreover, the performance of a given RDT varies depending on the setting in which it is being used. Dengvaxia is recommended for individuals age 9 years and above, primarily in endemic areas. If information about seroprevalence in the target population is available, measuring the probability that a positive test result is truly positive (positive predictive value, PPV), and a negative test result is truly negative (negative predictive value, NPV), can aid in determining acceptable levels of misclassification (12, 13). In 2019, the GDAC recommended RDTs have a PPV and NPV of ≥95%, with a minimum of ≥90% (12). Using samples that are reflective of the settings in which the RDT will be used is important when calibrating RDT positivity cutoff values.

Another challenge for the diagnostic performance of dengue RDTs is the target antigen: most dengue RDTs use DENV1-4 recombinant envelope (E) protein, a primary target for neutralizing antibodies. However, antibodies targeting the E protein are highly cross-reactive, especially those targeting the fusion loop, which is both immunodominant and highly conserved across flaviviruses (15, 16). In the evaluation by Sanofi Pasteur, cross-reactivity in dengue RDTs was observed for individuals with prior Zika virus (ZIKV) infection (14). Given the structural and antigenic similarity of DENV and ZIKV, their co-circulation in Asia, Africa, and the Americas, and the recent ZIKV pandemic in 2014-2017, dengue RDT cross-reactivity for ZIKV-positive samples is of concern (16). More importantly, since it is unknown whether individuals with a single prior ZIKV infection and no prior DENV infection will benefit from or be harmed by vaccination with Dengvaxia®, for the time being these individuals must be excluded from vaccination (17).

In this study, we evaluated a commonly used dengue RDT (SD BIOLINE Dengue IgG/IgM) and report for the first time the evaluation of new dengue and Zika RDTs developed by Excivion, which use a modified E antigen to distinguish previous DENV and ZIKV immunity. Further, we tested the effect of modifying various assay parameters to potentially improve performance for dengue pre-vaccination screening. We found quantitative evaluation can improve RDT performance, especially for the Excivion Zika RDT, but the SD BIOLINE Dengue IgG/IgM and Excivion Dengue RDT still require further development to be used as optimal dengue pre-vaccination screening assays.

## Methods

### SD BIOLINE Dengue IgG/IgM RDT

#### Test Design and Architecture

The SD BIOLINE Dengue IgG/IgM RDT (Bitrodiagnostico, Quito, Ecuador) detects anti-DENV E protein-specific IgG and IgM antibodies in serum or plasma. Sample anti-DENV IgG and IgM antibodies form a complex with recombinant DENV1-4 E proteins conjugated with a colloidal gold marker. As Ig-DENV E complexes move along the strip, they are captured by a corresponding mouse monoclonal anti-human IgG or IgM antibody (G or M line). Rabbit anti-DENV IgG are positioned at the control (C) line to confirm viability of gold marked DENV E proteins.

#### Clinical Samples and Ethical Statements

The IgG component of the SD BIOLINE Dengue IgG/IgM RDT was evaluated using samples from two cohorts. Blood samples (n=24) collected 1-30 years following confirmed DENV and/or ZIKV infection were provided by adults (age 18-93 years) recruited in Portland, Oregon with histories of travel to dengue-endemic areas **(** **Table 1**). Infection histories were confirmed by RT-PCR and/or focus reduction neutralization test (FRNT), as described previously (18). Signed consent was obtained from subjects prior to sample and data collection under a protocol approved by the Oregon Health & Science University Institutional Review Board (IRB) (IRB# 10212). In a separate study, serum samples (n=69) were provided by participants in the EcoDess cohort in Borbón, Ecuador **(** **Table 1** **)**. Participants ranged in age from 2-60 years old, with an even proportion of individuals of each sex. Samples were collected in October 2018, ~four months after the high DENV transmission season. DENV seroprevalence in this population was estimated to be 64%. DENV serostatus was measured using the Dengue PanBio IgG ELISA, using cutoff values established by DENV1-4 PRNT titers: DENV-naïve (<0.2 units), DENV-primary (0.2-0.9) and DENV-secondary (>0.9) (19). ZIKV serostatus was measured with the gold-standard ZIKV non-structural protein 1 blockade-of-binding assay (inhibition ≥50% was defined as positive) (20). The IRBs of the University of Michigan and the Universidad San Francisco de Quito approved the EcoDess Study. All individuals over age 18 provided informed consent. Parents provided informed consent for children under age 18, and all children age 6 and older also provided oral assent.

**Table 1:** Description of cohorts and samples used for evaluating each rapid diagnostic test.

Group	Sample characteristics	Origin	Age/seroprevalence	Confirmation test used
**Samples used to evaluate the SD BIOLINE Dengue IgG/IgM RDT**				
Oregon resident DENV immune cohort	Total **n=24**	Portland, Oregon USA	Adults (ages 18-93)	RT-PCR, Focus Reduction Neutralization Test
	(DENV-naïve = 5)			
	(DENV-positive = 16)			
	(ZIKV-positive = 3)			
	Collected 1-30 years post infection			
EcoDess cohort	Total **n=69**	Borbón, Ecuador	Children and adults (ages 2-60)	Dengue PanBio IgG ELISA
	(DENV-naïve = 21)		Seroprevalence: 64%	ZIKV non-structural protein 1 blockade-of-binding assay
	(DENV-positive = 44)			
	(ZIKV-positive = 4)			
	Collected ~4 months after high DENV transmission season			
**Samples used for preliminary evaluation of the Excivion Dengue RDT**				
SeraCare Dengue Mixed Titer Accuset™ Performance Panel	Total **n=12**	N/A	N/A	Multiple DENV ELISA and RDT tests
	(DENV-naïve = 1)			
	(DENV-positive = 11)			
	Recent and historic DENV infections			
Naval Medical Research Center	Total **n=30**	NAMRU-2 & NAMRU-6	Adults	Microneutralization, PRNT, RT-PCR
	(DENV-naïve = 9)			
	(DENV-positive = 18)			
	(ZIKV-positive = 3)			
	Acute and early convalescent samples (1-21 days post symptom onset)			
**Samples used to evaluate the Excivion Dengue RDT**				
ECUAVIDA Study	Total **n=115**	Quininde, Ecuador	Children (age 8)	Abcam DENV ELISA and ZIKV ELISA
	(DENV-naïve = 41)			
			Seroprevalence: 64%	
	(DENV-positive = 74)			
	Collected at annual routine follow-up visits			
Nicaragua Pediatric Dengue Cohort and Dengue Hospital-based Studies	Total **n=52**	Managua, Nicaragua	Children (ages 2-14)	DENV inhibition ELISA, ZIKV NS1 blockade-of-binding assay, RT-PCR and/or virus isolation, Neutralization assay
	(DENV-naïve = 5)			
			Seroprevalence in 2004: 86% 2015: 32%	
	(DENV-positive = 33)			
	(ZIKV-positive = 14)			
	Collected 6-12 months post infection			
**Samples used to evaluate the Excivion Zika RDT**				
Nicaragua Pediatric Dengue Cohort and Dengue Hospital-based Study Samples	Total **n=40**	Managua, Nicaragua	Children (ages 2-14)	ZIKV non-structural protein 1 blockade-of-binding assay, RT-PCR, Neutralization assay
	(DENV-positive = 10)			
	(ZIKV-positive = 30)			
	Collected 6-12 months post infection			

#### Test Evaluation and Data Collection

The SD BIOLINE Dengue IgG/IgM RDT was used as recommended by the manufacturer. Images were collected of the RDT strip at 0.25, 0.5, 0.75, 1, 1.5, 2, and 3 hours using a standardized light source with an iPhone 6 camera positioned 15cm above each test. IgG band positivity was evaluated visually by having independent analysts classify RDT results as negative (0), ambiguous (0.5), and positive (1). For the Oregon cohort, each sample was tested in two independent experiments, and a blinded analyst evaluated all images. For the EcoDess cohort, three independent, blinded analysts evaluated each image. We report average consensus values across analysts (values >0.5 are positive). Quantitative IgG band pixel intensity was measured from images using the EBImage package in R (21). Background pixel intensity of the RDT strip was subtracted from the IgG band. Higher values indicate stronger signal.

### Excivion Dengue RDT and Zika RDT

#### Test Design and Architecture

**Figure 1:**
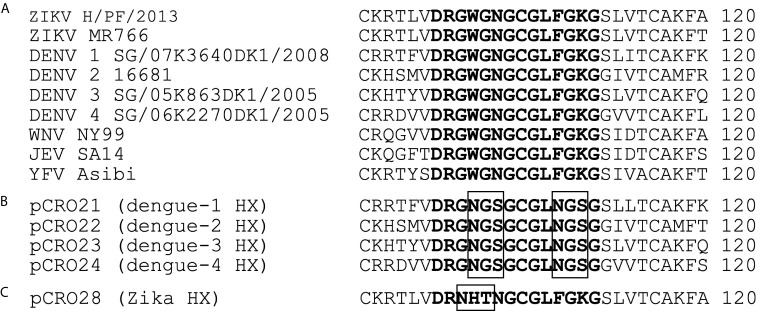
Sequence alignment of the highly conserved fusion loop region of the E proteins for distinct flaviviruses compared to the Excivion DENV1-4 and ZIKV fusion loop-glycosylated antigens. **(A)** Sequence homology between ZIKV, DENV1-4, West Nile virus, Japanese encephalitis virus, and yellow fever virus as previously described (15). Sequences of DENV1-4 **(B)** and ZIKV **(C)** used for the Excivion Dengue and Zika RDTs, with position of the N-linked glycosylation sequon boxed in black.

DENV1-4 and ZIKV E proteins were engineered to contain a sequence-programmed N-linked glycosylation of the fusion loop (US Patent Application No.16/303,588) **(** **Figure 1** **)**. The baculovirus-expressed fusion-loop hyperglycosylated exodomain E antigens (abbreviated FL-glycosylated E) were produced in Tni cells (US Patent Application No. 16/615,788). The primary DENV1-4 and ZIKV FL-glycosylated E antigen targets contained a C-terminally located hexahistidine tag to allow arrest at a line of monoclonal anti-His-tag antibody on the nitrocellulose test strip. The sample pad additionally contained an excess amount of non-His-tagged FL-glycosylated DENV or ZIKV E antigen to adsorb out residual cross-reactive antibodies directed at sites other than the fusion loop and block a false signal.

To construct each RDT, nitrocellulose (MDI Advance Microdevices, grade 150CNPH-N-SS40) was laminated onto 250 mm adhesive backing card (Lohmann) and striped with 0.3 mg/mL ReadyTag anti-6 His (BioXcell, cat. no. RT0266) as the test line and 1 mg/mL human IgG (Sigma-Aldrich, cat. no. 18640) with acid blue dye as the control line. The conjugate pad (Ahlstrom, grade 6615) was pre-treated in a solution containing 0.1 M Tris-HCl, pH 8.0, 0.5% bovine serum albumin and 0.25% Tween-20. Gold colloid (40 nm, BBI Solutions) was conjugated to anti-human IgG Mab (Eastcoast Bio, cat. no. HM295) at 3.5 µg/mL in 25 mM borate buffer, pH 9.0 followed by blocking by adding 10% bovine serum albumin in water at a final concentration of 0.5%. After washing and centrifugation the gold conjugate was resuspended to give an effective concentration of 10 gold units per microliter. The conjugate was sprayed using a nebulizer onto the previously prepared and dried conjugate pad so that there were 3.5 µl gold conjugate per test. For the DENV RDT, the sample pad (Ahlstrom, grade 8951) was striped with His-tagged DENV1–4 FL-glycosylated E antigens (25 ng of each per test) and and Strep-tag II ZIKV FL-glycosylated E antigen (1 µg per test) in PBS buffer (Sigma-Aldrich, cat. no. P4417) as a single line at 0.5 µl/mm. For the ZIKV RDT, the sample pad was striped with His-tagged ZIKV FL-glycosylated E antigen (50 ng per test) and Strep-tag-II DENV2 FL-glycosylated E antigen (10 µg per test) in PBS buffer at 1.5 µl/mm. In both RDTs, acid blue dye was added to the solution at 0.5% to facilitate visualization of the striping procedure. An absorbent pad (Whatman, CF5) was laminated to the distal end of the backing card with a 1-2 mm overlap with the previously laminated nitrocellulose. The gold-coated conjugate pad was laminated proximal to the nitrocellulose with a 1-2 mm overlap, and finally the antigen striped sample pad was positioned upstream of the conjugate pad with a 1-2 mm overlap. The fully laminated 250 mm cards were cut into 5 mm individual test strips using a Kinematic cutter and then assembled into plastic lateral flow cassettes. Tests were packaged in a foil pouch containing cassettes, desiccant, and a separate squeeze bottle of buffer and stored at 4° C in the dark until used.

#### Clinical Samples and Ethical Statements

The Excivion Dengue RDT was initially evaluated using samples from acute/convalescent confirmed dengue cases and recent infections. The first set of acute and early convalescent (days 1-21 post-symptom onset) deidentified clinical samples were used by the Naval Infectious Diseases Diagnostic Laboratory at the Naval Medical Research Center, and included samples provided by Naval Medical Research Unit Two (NAMRU-2) (Phnom Penh, Cambodia) and the Naval Medical Research Unit Six (NAMRU-6) (Lima, Peru) under approved human use protocol #: PJT-17-06 **(** **Table 1** **)**. Past infection history and/or serostatus was determined by CDC Trioplex real-time RT-PCR and dengue IgM and IgG ELISAs. ELISA-positive samples were tested using the CDC dengue PRNT against DENV2, DENV3, and DENV4 (positive defined as PRNT_90_ titer of ≥10) or DENV microneutralization tests as previously described (22). A separate independent evaluation was completed using the SeraCare Dengue Mixed Titer AccuSet™ Performance Panel (0845-0074), which consisted of 21 single collection-event serum samples (anti-DENV IgG positive, IgG and IgM positive, and negative) from multiple individuals in Honduras and India (**Table 1**). An informed consent form was signed by the donor, the donor’s next of kin, and/or an authorized agent or a legal agent in accordance with applicable laws and regulations.

Full evaluation of the Excivion Dengue and Zika RDT was performed with samples from two endemic pediatric cohorts in Managua, Nicaragua at the University of California, Berkeley. The dengue serum/plasma set (n=52) and the Zika set (n=40) were from children 2-17 years of age (n=74 unique samples) who had confirmed dengue or Zika cases (n=5 had only neutralization-assay confirmed inapparent infections) and for whom likely prior DENV and ZIKV infections could be determined (17, 23, 24) **(** **Table 1** **)**. Children in the community-based cohort were followed longitudinally as they experience their first and subsequent DENV and ZIKV infections (25, 26). In the hospital-based study, children enroll upon disease presentation (27). All samples were collected 6-12 months post-infection. Infecting virus was confirmed by RT-PCR, virus isolation, or a serological algorithm for the small subset of PCR-negative cases (23). DENV serostatus and DENV infections were confirmed by testing paired annual samples by the DENV inhibition ELISA and/or reporter-virus based neutralization assay (6, 28, 29). ZIKV serostatus and ZIKV infections were measured in paired annual samples using the ZIKV inhibition ELISA and ZIKV NS1 BOB (20). Individuals were classified as naïve if they entered the study DENV-seronegative and experienced no infection events. Primary versus secondary DENV infection was evaluated based on the number of observed prior DENV infections or confirmed dengue cases in the cohort study or by confirmed dengue case and convalescent anti-DENV inhibition ELISA titer (>1:2560, based on cutoffs determined for the gold-standard hemmaglutination inhibition assay) in the hospital study (27, 28). Seroprevalence in children 9 years of age in this population (the age of eligibility for Dengvaxia) was 86.6% in 2004 but dropped over time to 32.2% by 2015 (30). The Nicaragua Pediatric Dengue Cohort Study and Pediatric Dengue Hospital-based Study were reviewed and approved by the institutional review boards of the University of California, Berkeley, and the Nicaraguan Ministry of Health. Parents or legal guardians of all subjects provided written informed consent, and subjects ≥6 years old provided assent.

The Excivion Dengue RDT was also tested using a representative set of serum samples collected between August 2014 and December 2016 from 115 healthy children 8 years of age participating in routine visits in the ECUAVIDA birth cohort in the Quininde district in Esmeraldas, Ecuador **(** **Table 1** **)**. DENV seroprevalence for children 6-10 years of age in this population was 64-67% in 2015 (31). Zika emerged in Ecuador in late 2015 and caused a nation-wide epidemic in the spring of 2016 (n=18 samples were collected after the emergence of Zika). DENV serostatus was measured using the Abcam Human Anti-Dengue virus IgG ELISA Kit (ab108728). ZIKV serostatus was measured using the Abcam anti-Zika virus IgG ELISA kit (ab221844) and the anti-Zika virus IgM ELISA kit (μ-capture) (ab213327) (Abcam, Cambridge, UK). None of the samples collected before the emergence of Zika were ZIKV-IgG positive (0/11 tested) while 14/18 collected after the emergence of Zika were positive. The IRBs of the Hospital Pedro Vicente Maldonado and the Universidad San Francisco de Quito approved the study protocol. Informed written consent was obtained from a parent and minor assent from the child.

#### Test Procedure and Data Collection

Serum/plasma (5 µL) was added to the sample port and incubated 1-5 minutes at room temperature. Tests were chased with 120 µL of running buffer containing PBS, 0.025% Tween-20 and 0.5% polyethylene glycol, 20K (Sigma-Aldrich). All tests were run at room temperature for 0.5 hours before visual and quantitative data collection. For the Nicaragua samples, images of the test strip were also collected using the iPhone 6 by 1-3 operators and IgG band intensity was also measured at 0.75 and 1 hours. To evaluate reproducibility for the Nicaragua samples, 10 samples were run independently in separate tests and a subset of tests was measured twice using the ChemBio Cube portable lateral flow test reader.

#### Test Evaluation and Data Collection

Qualitative IgG band positivity was evaluated independently by SeraCare Life Sciences (now LGC) for the Dengue Mixed Titer Accuset™ Performance Panel. For the NMRC samples, the tests were performed and evaluated blindly in duplicate by two independent operators. Discrepancy in result interpretation was resolved using a third independent observer. Analysts assigned band intensity according to a scale from 0-5 (0= no band to 5= very strong band). The average value is reported. For the Nicaragua samples, one operator conducted all tests. Two independent analysts qualitatively evaluated IgG band positivity, with each reporting a value of 0 for negative, 0.5 for ambiguous, and 1 for a positive test result. The average value was reported and values >0.5 were considered positive and ≤0.5 as negative. *Quantitative* IgG band positivity for the ECUAVIDA and Nicaragua study samples was calculated using the ChemBio Cube portable lateral flow test reader. Higher values indicate stronger signal.

### Data Visualization and Statistical Analyses

Individual samples were grouped by shared DENV and ZIKV infection histories based on the reference and/or gold-standard assays used for each cohort. All individual datapoints are plotted, with median and interquartile range intervals presented as summary statistics. Sensitivity and specificity were evaluated by comparing all DENV-seronegative samples (including primary ZIKV) to all DENV-seropositive samples. For some cohorts, we performed a secondary analysis comparing all DENV-negative samples only to primary DENV-positive samples. Sensitivity and specificity for visual measures of IgG band positivity were evaluated using contingency tables in GraphPad Prism 8 software. P-values and statistical significances were computed using Fisher’s exact test. For quantitative measures of IgG band positivity, pixel intensity positivity cutoff values and confidence intervals were derived using receiver-operating characteristic (ROC) curves using the hybrid Wilson/Brown method at each timepoint using R and Prism. Pixel intensity positivity cutoff values were then selected according to the GDAC optimal (≥98%) or minimum (≥90%) specificity (12). All sensitivity and specificity values at each timepoint were plotted in Prism and fit curves were generated using smoothing spline curves or simple linear regression when ≤3 timepoints were analyzed. PPV and NPV were calculated using the seroprevalence estimates for the corresponding age group and time of collection of samples in each cohort.

## Results

### SD BIOLINE Dengue IgG/IgM RDT

**Figure 2:**
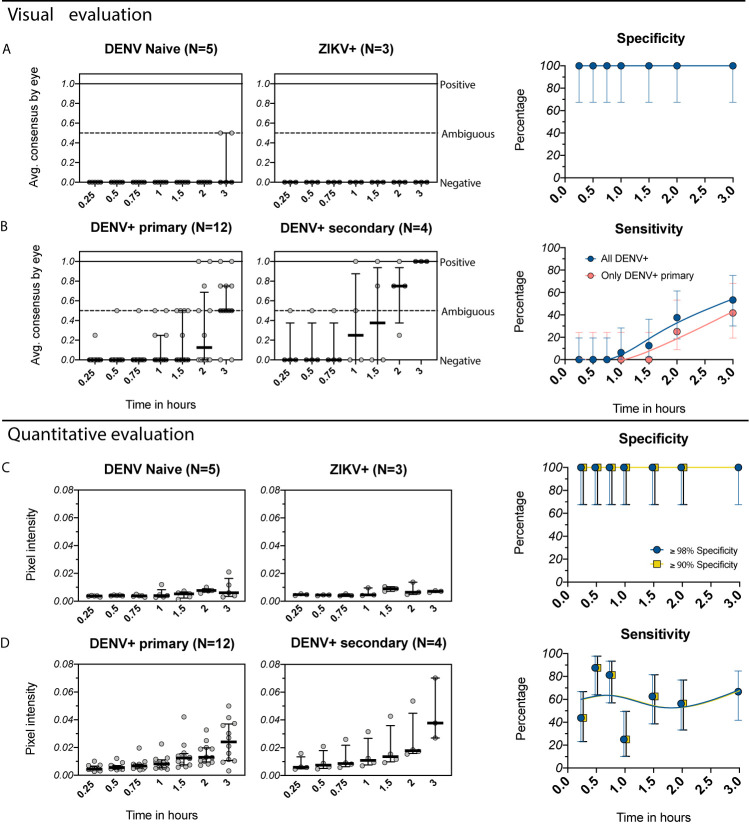
Evaluation of the IgG component of the SD BIOLINE Dengue IgG/IgM RDT using samples from the Oregon traveler cohort. **(A, B)** Visual readings for IgG band positivity evaluated in two independent experiments (average consensus value shown as individual dots) at multiple timepoints. Medians and interquartile ranges are shown as summary statistics. Data are plotted separately for **(A)** DENV-naïve (flavivirus naïve or yellow fever vaccine recipients) and ZIKV+ (prior ZIKV infection but not DENV infection) samples and **(B)** DENV+ primary (primary DENV infection with one of the four serotypes) and DENV+ secondary (multiple prior DENV infections or prior DENV and ZIKV infections). Corresponding specificity and sensitivity values (dots) are shown for analyses using all DENV-positive samples (blue) versus only primary DENV-positive samples (pink) with 95% confidence intervals (vertical bars) for each timepoint. Smoothing spline curves are used to visualize trends over time. **(C, D)** Equivalent to A and B, but with quantitative evaluation of RDT tests performed using R, measured as IgG band pixel intensity. Data again are grouped by infection history and timepoint. Specificity was set to the optimal value (≤98%) or minimum value (≤90%) recommended by GDAC, although for this dataset, the only specificity ≤90% was 100% and thus the same value is shown for both analyses; corresponding sensitivity values are shown.

We first evaluated the performance of the SD BIOLINE Dengue IgG/IgM RDT to detect prior DENV infections in travelers recruited in Portland, Oregon who experienced virologically (RT-PCR) and/or serologically (FRNT) confirmed DENV and ZIKV infections while traveling to an endemic area. Serum from DENV-naïve individuals who had not visited endemic areas were also tested. The RDT was performed as recommended by the manufacturer, with test positivity evaluated visually at 0.25 hours. We found that the 5 DENV-negative individuals (2 yellow fever vaccinee recipients and 3 individuals with one prior ZIKV infection, ZIKV+) were consistently negative **(** **Figure 2A** **)**, yielding a test specificity of 100%. However, none (0/16) of the individuals with history of one (DENV+ primary) or multiple prior DENV infections (DENV+ secondary) were detected by the SD BIOLINE Dengue IgG/IgM RDT, yielding a sensitivity of 0% **(** **Figure 2B** **)**.

In preliminary analyses using control sera, we had observed that the IgG line turned positive in some samples when tests were allowed to run for longer than recommended. We tested the hypothesis that detection of historical DENV infections would improve with time by reading the test at serial intervals of 0.25, 0.5, 0.75, 1, 1.5, 2, and 3 hours. All DENV-negative samples remained negative at all timepoints (specificity: 100%) **(** **Figure 2A** **)**. However, even at later timepoints, only a subset of DENV+ primary samples (5/12) were positive, with differences in positivity by serotype (lower for DENV1 and DENV4 than DENV2 and DENV3, **Figure S1**). Sensitivity for this group was 0% between 0.25 and 1.5 hours and only reached 42% when tests were read at 3 hours **(** **Figure 2B** **)**. Overall, sensitivity including all DENV-positive samples increased at later timepoints but only reached 53% percent by 3 hours **(** **Figure 2B** **)**.

As an alternative approach to improve dengue RDT performance, we tested whether measuring IgG intensity quantitatively would enable identification of optimal cutoff values for distinguishing positive from negative samples. Band pixel intensity was measured from images of the test strip using R. Using this approach, DENV-negative samples maintained lower band intensity values overall, whereas we detected a continuous increase in band intensity over time for samples with a history of DENV infection **(** **Figures 2C, D** **)**. Quantitative evaluation enables selecting positivity cutoff values based on assay performance criteria, and for dengue pre-vaccination screening, specificity should be prioritized. Thus, we evaluated sensitivity after setting the specificity according to the GDAC recommended optimum (≤98%) and minimum (≤90%). (In this instance, the only specificity ≤90% was 100% and thus the same value is shown for both specificity cutoffs.) Using this approach, for both specificity levels, sensitivity was much higher at early timepoints (44% at 0.25 hours, 88% at 0.5 hours, 81% at 0.75 hours) than when evaluated visually (0%) **(** **Figure 2D** *vs.* **2B** **)**. Although still below the desired sensitivity of 95% proposed by GDAC, this demonstrates the potential benefit of using quantitative readers to maximize detection of DENV-positive individuals.

**Figure 3:**
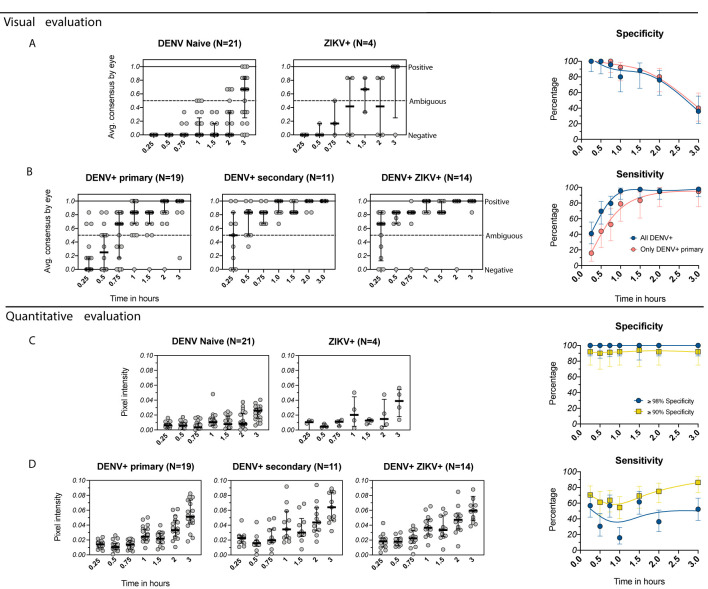
Evaluation of the IgG component of the SD BIOLINE Dengue IgG/IgM RDT using samples from the EcoDess cohort in Esmeraldas, Ecuador. **(A, B)** Average visual readings for IgG band positivity evaluated in one experiment by three separate analysts at multiple timepoints (dots show individual points, groups are summarized by medians and interquartile ranges). Data are plotted separately for **(A)** DENV-naïve and ZIKV+ only samples and **(B)** DENV-positive only with either primary or secondary DENV immunity, or DENV+ and ZIKV+ samples. Corresponding specificity and sensitivity values (dots) are shown for analyses including all DENV-positive samples and only primary DENV-positive samples, with 95% confidence intervals (vertical bars) for each timepoint. Specificity and sensitivity values are shown to the right, with estimates fitted using smoothing splines to visualize trends. **(C, D)** Equivalent to **(A, B)**, but with quantitative evaluation of RDT tests using R, measured as IgG band pixel intensity. Specificity was set to optimum (≥98%) and minimum (≥90%) values, and corresponding sensitivity values are shown.

To evaluate the performance of the SD BIOLINE IgG/IgM RDT in a DENV and ZIKV-endemic setting, we tested healthy samples collected during the low-DENV transmission season from 69 individuals ages 2-60 participating in a dengue and Zika cohort study in Esmeraldas, Ecuador. Given that no gold-standard was available, DENV serostatus was determined using the PanBio DENV IgG ELISA using cutoff values established for this assay using the FRNT as previously described (19). ZIKV serostatus was defined using the ZIKV NS1 BOB assay, an assay that has been extensively evaluated and is highly specific (20). When each RDT was evaluated visually, DENV-seronegative samples (n=25) were negative at earlier timepoints but became ambiguous after 1-2 hours (**Figure 3A**), with specificity dropping from 100% at 0.25-0.5 hours to 80% at 1 hour. DENV-seropositive samples (n=44) became more positive over time (**Figure 3B**), with sensitivity of 41% at 0.25 hours (primary only: 16%) but reaching 96% when read at 1 hour (primary only: 79%). When instead the RDT was evaluated quantitatively, specificity values could be maintained at the levels recommended by GDAC (≥98% and ≥90%) while sensitivity at 0.25 hours remained high (57% and 70%, respectively) (**Figures 3C, D** **)**. Sensitivity varied with time beyond 0.25 hours but was relatively stable and above 55% when using the minimum specificity ≥90%.

Because this cohort is based in an endemic area where dengue vaccines might be used, we were also able to consider the performance of the assay with respect to local seroprevalence, which was used to estimate PPV and NPV. The PPV was 100% at ≥98% specificity and was 92% at ≥90% specificity, reaching the optimal 95% at 1.5 and 3 hours **(** **Figure S2-A** **).** NPV was higher when using optimal specificity (≥98%) and peaked at 1 hour (87%) but never reached the minimum of 90%, as recommended by GDAC. Overall, when tests were evaluated visually, sensitivity increased with time at the expense of specificity, limiting reading of the RDT to earlier timepoints. Quantitative evaluation enabled the tests to reach recommended specificity and PPV, while also improving sensitivity and NPV.

### Excivion Dengue RDT

Other flaviviruses can induce antibodies that target the conserved E protein fusion loop and result in false positive dengue RDT results. Such cross reactivity became an urgent concern after the Zika epidemic, given the close antigenic relatedness of DENV and ZIKV (16). To address the need for a dengue RDT that sensitively and specifically detects remote prior DENV infection for use in a mass dengue vaccination campaign, Excivion developed a novel lateral flow dengue RDT using DENV1-4 E proteins in which two asparagine-linked glycans were inserted into E fusion loop **(** **Figure 1** **).** The glycan blocks antibodies from binding the conserved fusion loop without disrupting the overall structure of the E protein, leaving other E epitopes accessible. Further modifications were made to the test design to overcome cross-reactivity, including incorporation of excess glycan-blocked ZIKV E protein to preabsorb cross-reactive antibodies to epitopes outside the fusion loop. We first evaluated how the Excivion Dengue RDT performed as a standard diagnostic assay using acute and convalescent samples from confirmed dengue and Zika cases (1-21 days post symptom onset) tested at the Naval Infectious Diseases Diagnostic Laboratory at the Naval Medical Center. Infections were confirmed by RT-PCR and IgM and IgG ELISA. All ELISA-positive samples were further tested using either the CDC PRNT against DENV2, DENV3, and DENV4 (positive: PRNT_90_ titer of ≥10) or DENV microneutralization tests. None of the 12 DENV-negative samples were positive by the Excivion Dengue RDT, yielding a specificity of 100%. Notably, 3 primary ZIKV infection samples, which were negative by DENV neutralization test, were also negative in the Excivion Dengue RDT. Further, the Excivion dengue RDT detected 17 out of 18 DENV-positive samples for an overall sensitivity of 94% (**Table 2**). The performance of the Excivion dengue RDT was then independently compared to other dengue IgG ELISAs and RDTs using the SeraCare Dengue Mixed Titer Accuset™ Performance Panel (n=11). The Excivion dengue RDT had higher sensitivity (100%) for detecting samples likely to have had recent (IgM and IgG) or historic (IgG only) DENV infections compared to the SD BIOLINE Dengue Duo RDT (18%) and the PanBio IgG RDT (9%) (**Table 3**). Further, the Excivion dengue RDT was as sensitive for detecting DENV-seropositive samples as five commercially available DENV IgG ELISAs **(** **Table 3**).

**Table 2:** Preliminary evaluation of the Excivion Dengue RDT to classify acute and early convalescent DENV and ZIKV infection samples.

Sample ID	Sample confirmation	Infection history	DENV RDT score	ZIKV PRNT 90	DENV Neut titer
28523	NHS	Naïve	0	<10	<20^a^
28696	NHS	Naïve	0	<10	<20^a^
28697	NHS	Naïve	0	<10	<20^a^
28962	NHS	Naïve	0	<10	<20^a^
1183	PCR-/DENV ELISA-	Naïve	0	ND	ND
1184	PCR-/DENV ELISA-	Naïve	0	ND	ND
1208	PCR-/DENV ELISA-	Naïve	0	ND	ND
1213	PCR-/DENV ELISA-	Naïve	0	ND	ND
1214	PCR-/DENV ELISA-	Naïve	0	ND	ND
PT1-2017	PCR ND/PRNT+	ZIKV+	0	160	<10
778	PCR-/PRNT+	ZIKV+	0	78	<10
530	PCR-/PRNT+	ZIKV+	0	266	<10
574	PCR-/PRNT+	DENV+ZIKV+	0	358	10
970	PCR-/PRNT+	DENV+ZIKV+	1	217	>80
389	PCR-/PRNT+	DENV+ZIKV+	1.5	25	>80
29430	NHS	DENV+ZIKV+	2	20	320^a^
555	PCR-/PRNT+	DENV+ZIKV+	3	118	>80
375	PCR-/PRNT+	DENV+ZIKV+	3.5	214	>80
1136	PCR-/PRNT+	DENV+ZIKV+	4	40	>80
6219c	DEN1	DENV+ZIKV+	4	>320	10240^a^
21203c	DEN1	DENV+ZIKV+	4.5	10	5120^a^
FPI04237	DEN2	DENV+ZIKV+	4.5	10	40960^a^
FPI05679	DEN2	DENV+ZIKV+	4.5	10	81920^a^
FPI05163	DEN2	DENV+ZIKV+	4.5	20	81920^a^
1184c	DEN1	DENV+ZIKV+	4.5	80	10240^a^
618	PCR-/PRNT+	DENV+ZIKV+	4.5	492	>80
FPI10111	DEN2	DENV+	5	<10	20480^a^
6720c	DEN1	DENV+ZIKV+	5	40	10240^a^
21702c	DEN1	DENV+ZIKV+	5	80	20480^a^
FPI10014	DEN2	DENV+ZIKV+	5	>80	>162840^a^
Sensitivity			17/18 (94%)		
Specificity			12/12 (100%)		

a DENV microneutralization test, otherwise DENV2-4 PRNT_90_.

**Table 3:** Preliminary evaluation using the SeraCare™ Panel showing the performance comparison of dengue E protein IgG ELISAs and E protein IgG RDTs to the Excivion Dengue RDT.

Sample type	PanBio IgG ELISA	EURO-IMMUN IgG ELISA	Calbio-tech IgG ELISA	Standard Diagnostics IgG ELISA	Focus DxSelect IgG ELISA	SD BIOLINE DengueDuo IgG RDT	PanBio IgG RDT	Excivion Dengue IgG RDT
Recent DENV infection (IgM & IgG)	6.12	4.77	1.83	7.87	9.65	+	+	+++
	5.07	4.51	1.64	7.87	8.54	+	–	+++
	4.47	4.16	1.74	7.87	8.03	–	–	+++
	0.75	3.10	1.74	0.89	4.48	–	–	++
Historic DENV Infection (IgG only)	3.46	3.81	1.62	3.55	7.35	–	–	+++
	1.85	3.50	1.94	2.67	7.52	–	–	+++
	1.48	3.83	1.85	2.37	7.57	–	–	+++
	1.04	3.77	2.07	1.54	6.93	–	–	+++
	0.37	1.17	1.32	0.62	2.12	–	–	+
	0.39	1.16	1.09	0.41	4.22	–	–	+++
	0.28	2.97	1.84	0.91	4.89	–	–	+
DENV-naïve	0.05	0.26	0.25	0.23	0.43	–	–	–
Sensitivity						2/11 (18%)	1/11 (9%)	11/11 (100%)

“-” = negative.

“+” = low positivity.

“++” = medium positivity.

“+++” = high positivity.

**Figure 4:**
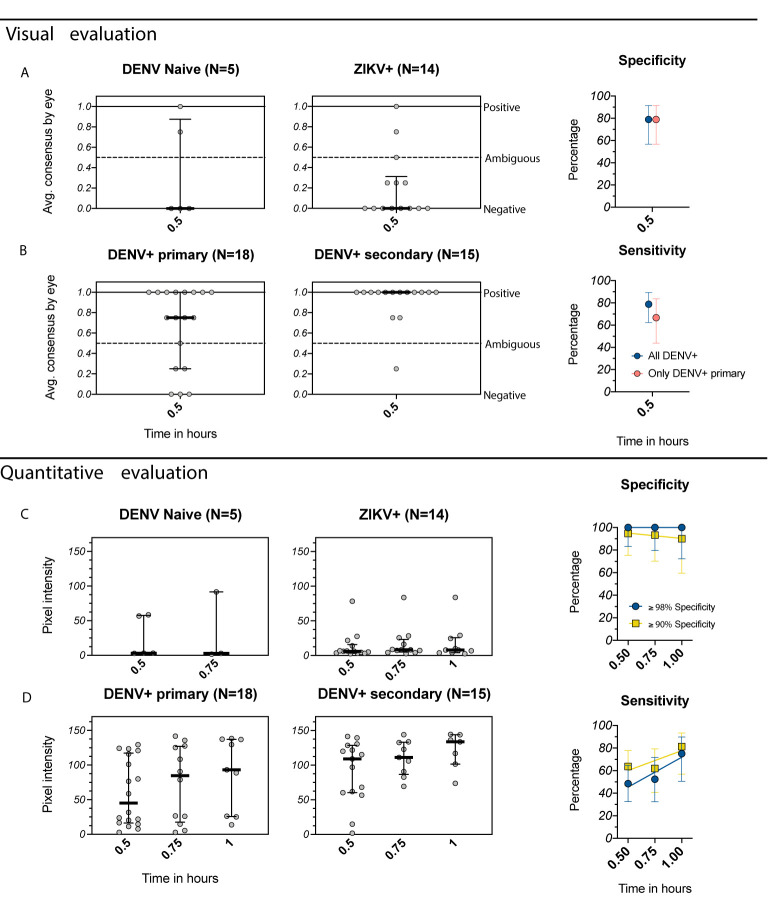
Evaluation of the Excivion Dengue IgG RDT using samples from the Nicaragua Pediatric Dengue Cohort and Hospital-based Studies. **(A, B)** Visual evaluation of the IgG band positivity in one experiment by two separate analysts (individual dots). Data are grouped by DENV and ZIKV infection history (medians and interquartile ranges), separated by **(A)** DENV-naïve and ZIKV+ samples and **(B)** DENV+ primary and DENV+ secondary samples (including multiple prior DENV and/or ZIKV infections). Corresponding specificity and sensitivity values (dots) are shown for both analyses of all DENV-positive samples versus only primary DENV-positive samples. Vertical bars show 95% confidence intervals for each timepoint. **(C, D)** Equivalent to A and B, but with quantitative evaluation of RDT tests using the ChemBio Cube lateral flow reader, measured as IgG band pixel intensity at multiple timepoints. Simple linear regressions are used to visualize trends over time. Optimal (≥98%) and minimum (≥90%) specificity and corresponding sensitivity values are shown.

We performed a full evaluation of the Excivion Dengue RDT using samples collected six or more months after confirmed DENV and ZIKV infections from children participating in pediatric cohort and hospital studies in Managua, Nicaragua. Dengue and Zika cases were confirmed by RT-PCR and/or virus isolation, with a subset confirmed by serological algorithm. Inapparent DENV and ZIKV infections were confirmed by DENV and ZIKV iELISA, DENV neutralization assay, and/or ZIKV NS1 BOB on paired annual samples. Most samples were from children with primary DENV and ZIKV infections (n=32), the most difficult samples to classify. When evaluated visually at 0.5 hours, 3/5 DENV-naïve and 12/14 primary ZIKV infection samples were negative by the Excivion Dengue RDT, with a total specificity of 79% (**Figure 4A**). Additionally, 12/18 primary DENV samples were positive (sensitivity of 67%) and 14/15 secondary DENV infection samples were positive, for a total sensitivity of 79% (**Figure 4B**). Sensitivity also differed by serotype, with the highest sensitivity for DENV2 (**Figure S3**). When the Excivion Dengue RDT was evaluated quantitatively and specificity was set to ≥98%, sensitivity reached 49% at 0.5 hours and further increased with time (52% at 0.75 hours, 75% at 1 hour) (**Figures 4C, D** **)**. At a specificity of ≥90%, sensitivity was high and further increased with time, reaching 81% at 1 hour. We also considered the performance of the Excivion Dengue RDT in the context of DENV seroprevalence in 9-year-old children in Nicaragua, which dropped from 86.6% in 2004 to 32.2% by 2015 (30). When seroprevalence was high, PPV was high (100%), but NPV was lower across timepoints and specificities (29%-68%). When seroprevalence was lower, PPV and NPV values remained the same when specificity was set to ≥98%, but PPV dropped (<85%) while NPV rose (84-91%) at specificities ≥90% (**Figure S2-B**). Overall, the Excivion Dengue RDT had lower than ideal specificity when evaluated visually but was able to achieve high sensitivity, including when evaluated quantitatively and when requiring high specificity.

**Figure 5:**
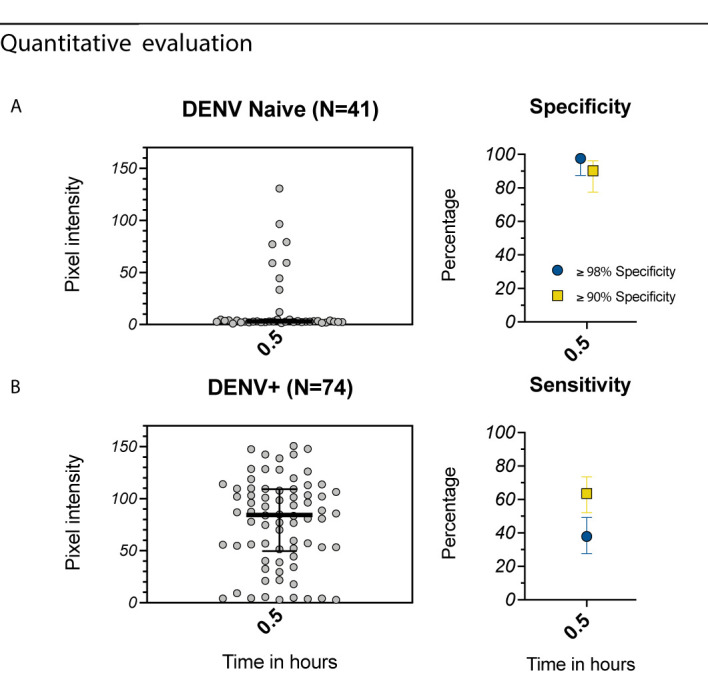
Evaluation of the Excivion Dengue IgG RDT using samples from the ECUAVIDA Study. **(A, B)** Quantitative evaluation of RDT tests using the ChemBio Cube lateral flow reader, measured as IgG band pixel intensity (medians and interquartile ranges are shown as summary statistics). Data are plotted separately for **(A)** DENV-seronegative and **(B)** DENV-seropositive samples as classified by Dengue Abcam ELISA. Specificity and sensitivity values (dots) with 95% confidence intervals (vertical bars) are shown at a single timepoint, with specificity set to either ≥98% or ≥90%. Corresponding sensitivity values are shown.

We further evaluated the Excivion Dengue RDT in a cohort of healthy eight-year-old children participating in the ECUAVIDA cohort in Esmeraldas, Ecuador. The cohort is representative of the population where Dengvaxia might be used, with seroprevalence of 64-67%. Because no gold-standard assays were available, DENV seropositivity was measured using the Abcam dengue ELISA, while ZIKV positivity was measured using the Abcam ZIKV ELISA. When evaluated quantitatively and at a specificity of ≥90%, the Excivion dengue RDT had a sensitivity of 64%, while at a specificity of ≥98%, sensitivity was 38% (**Figure 5**). However, at ≥98% specificity, PPV was 97% and NPV 72%, surpassing the PPV recommended by GDAC (**Figure S2-C**). Overall, the Excivion Dengue RDT had lower than ideal sensitivity when detecting remote prior DENV infections but had high PPV and NPV within the seroprevalence context of this cohort.

### Excivion Zika RDT

**Figure 6:**
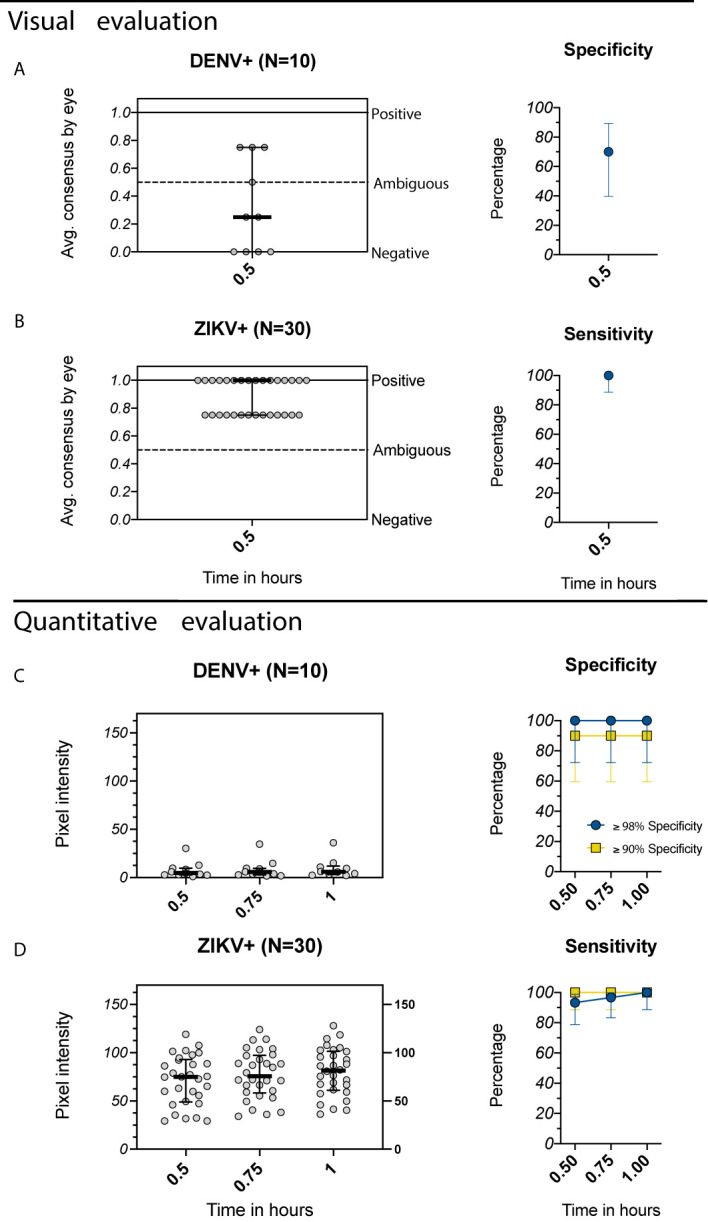
Evaluation of the Excivion Zika IgG RDT using samples from the Nicaragua Pediatric Dengue Cohort and Hospital-based Studies. **(A, B)** Average visual readings for IgG band positivity evaluated in one experiment by two separate analysts (individual dots) at single timepoint (medians and interquartile ranges are shown). Analyses are grouped by **(A)** DENV+ samples (primary DENV infection with one of the four serotypes or multiple prior DENV infections) and **(B)** ZIKV+ samples (prior ZIKV infection but not DENV infection). Corresponding specificity and sensitivity values (dots) with 95% confidence intervals (vertical bars) are shown. **(C, D)** Equivalent to A and B, but with quantitative evaluation of RDT tests using the ChemBio Cube lateral flow reader, measured as IgG band pixel intensity at multiple timepoints. Optimal (≥98%) and minimum (≥90%) specificity and corresponding sensitivity values are shown. Simple linear regressions show temporal trends.

Individuals with primary ZIKV immunity are at increased risk of future symptomatic and severe dengue disease (17). However, it is not yet known whether individuals with primary ZIKV immunity and no prior DENV infection will benefit from DENV vaccination, as it is possible that Dengvaxia could further increase their future risk of severe dengue (32). Because dengue RDTs can be affected by cross-reactive immunity induced by ZIKV, for DENV-ambiguous results, it may be valuable to simultaneously test for ZIKV-specific antibodies using a separate assay. We evaluated the Excivion Zika RDT, which uses a similar design to the Excivion Dengue RDT, with sera from children with well-characterized prior ZIKV and DENV infections in the same pediatric studies in Managua, Nicaragua. When the Excivion Zika RDT was evaluated visually at 0.5 hours, 3/10 samples classified as having prior DENV infections were positive by the Excivion Zika RDT, for a specificity of 70% (**Figure 6A**). However, all 30/30 samples classified as ZIKV-positive were positive by the Excivion Zika RDT, with a sensitivity of 100% (**Figure 6B**). When evaluated quantitatively, sensitivity increased from 93% at 0.5 hours to 100% at 1 hour (**Figure 6D**) while maintaining optimal (≥98%) specificity (**Figures 6C, D**). At minimum specificity (≥90%), sensitivity was perfect at all timepoints (100%).

## Discussion

Currently, there is not a licensed assay that can quickly, easily, and accurately measure DENV immunity in the context of a mass vaccination program. In this study, we evaluate the performance of the SD BIOLINE Dengue IgG/IgM RDT to detect remote prior DENV infections using non-endemic and endemic samples. We also provide the first report of dengue and Zika RDTs developed by Excivion, present their novel design, and evaluate their performance. To try to improve sensitivity and specificity, we further analyzed the effect of developing RDTs for longer than the recommended time and evaluating RDT positivity using quantitative readers. We found that when RDTs were evaluated visually and developed for longer periods of time, increases in sensitivity were offset by decreases in specificity. In contrast, with quantitative evaluation, specificity values meeting the GDAC criteria (≥98% or ≥90%) could be ensured while also achieving higher sensitivity for detecting endemic samples for all three RDTs studied.

Most existing dengue RDTs were designed to detect acute and recent DENV infections in symptomatic patients and thus are calibrated to facilitate disease diagnosis in populations with high prevalence of anti-DENV immunity. Using samples from both endemic and non-endemic areas, we found that an existing dengue RDT, the SD BIOLINE Dengue IgG/IgM RDT, performed poorly when used as a screening assay for DENV serostatus in healthy subjects. The RDT was highly specific (100%) when used as directed (0.25 hours, visually) but had low sensitivity (41%) for detecting DENV seropositivity in endemic samples and even lower sensitivity for detecting primary DENV immunity in endemic samples (16%) and primary or secondary DENV immunity in non-endemic samples (0%). If the SD BIOLINE Dengue IgG/IgM RDT were used as directed, individuals with primary DENV immunity, who are at high risk of future dengue disease and may benefit most from vaccination, would rarely test positive and receive the vaccine (6, 33). When the SD BIOLINE Dengue IgG/IgM RDT was developed up to 3 hours and evaluated visually, sensitivity increased with time at the expense of specificity. Because DENV vaccinations are earmarked for seropositive children in endemic areas, the inherent trade-off between sensitivity and specificity suggests that visual evaluation of the SD BIOLINE Dengue IgG/IgM RDT in endemic settings should be limited to earlier timepoints (before 0.5 hours). However, when tests were evaluated quantitatively, specificity could be set to ≥98% and still reach higher sensitivity (57%) at 0.25 hours, demonstrating the potential value of using RDT readers to maximize detection of DENV-seropositive individuals.

Aware of the drawbacks of current dengue RDTs, Excivion designed dengue and Zika RDTs to increase sensitivity and reduce cross-reactivity to other flaviviruses. Excivion aimed to eliminate recognition of the cross-reactive fusion loop and used preabsorption with excess off-target antigen to reduce noise from cross-reactive epitopes outside the fusion loop. In preliminary studies, we showed the Excivion Dengue RDT had high sensitivity (>94%) and specificity (100%) for detecting acute and early convalescent DENV- and ZIKV-positive samples. When visually evaluated with samples from children living in DENV and ZIKV-endemic Nicaragua and Ecuador with remote prior DENV infections, sensitivity and specificity values were equivalent, at 79%. However, quantitative evaluation of the Excivion Dengue RDT enabled prioritization of specificity, and when specificity was set to ≥98%, sensitivity was high (49%) and further increased over time. This observation suggests that alternative cutoff values may make this assay a strong candidate pre-vaccination screening assay, among the set of assays currently available. Further, use of quantitative RDT readers developed for a smartphone or tablet could further aid in improving the performance of this and other assays, making RDTs more like an ELISA in low resource settings (34). We also observed that the Excivion Dengue RDT had high PPV (>97%) in high DENV seroprevalence settings, although in low DENV seroprevalence settings, PPV dipped below target levels (<85%) when specificity was set only to ≥90%, as is expected (12, 13). These observations further underscore the importance of prioritizing high specificity for pre-vaccination screening assays, as population seroprevalence is dynamic and can fluctuate over time.

It is not yet known whether individuals with primary ZIKV immunity and no prior DENV infection will benefit from DENV vaccination, as it is possible that Dengvaxia or other upcoming dengue vaccines could increase future risk of severe dengue disease in this group (26, 32). We found the Excivion Zika RDT sensitivity was high (100%) at all timepoints when evaluated qualitatively but specificity was low (70%). However, sensitivity was high (>93%) and increased with time when evaluated quantitatively while keeping optimal specificity (≥98%). Because the performance of dengue RDTs can be affected by cross-reactive immunity induced by ZIKV, these results demonstrate a potential added value of additional testing with a Zika RDT, which could help identify those with only prior ZIKV immunity.

Our study has limitations. While many of our analyses of RDT sensitivity and specificity were compared to gold-standard assays (RT-PCR and/or neutralization assay), the EcoDess and ECUAVIDA cohorts were limited to classification of serostatus by the PanBio or Abcam Indirect IgG ELISA. A recent study comparing the PanBio dengue IgG ELISA to the FRNT found high correspondence, with a specificity of 93% and sensitivity of 95% for detecting DENV serostatus in a pediatric cohort in the Philippines (19). Thus, while a small proportion of the samples in the EcoDess and ECUAVIDA cohorts were likely misclassified, these studies were included because they are representative of the target populations for dengue vaccination and enabled estimation of seroprevalence, making it possible to calculate PPV and NPV.

Overall, our evaluation of the SD BIOLINE Dengue IgG/IgM RDT and first report of the novel Excivion dengue and Zika RDTs reveal that modifications to these assays can enable them to meet some but not all of the performance criteria set for dengue pre-vaccination assays. The modifications that we tested, including use of unique antigens/epitope targets in the Excivion RDTs, extension of RDT development time, and application of quantitative readers, show promise, improving sensitivity while maintaining high specificity, but likely require further optimization prior to use in dengue vaccination efforts. The development of better dengue RDTs remains a high priority for the currently licensed dengue vaccine and may be important for future dengue vaccination efforts.

## Data Availability Statement

The raw data supporting the conclusions of this article will be made available by the authors, without undue reservation.

## Ethics Statement

Oregon Health & Science University Institutional Review Board (IRB# 10212) approved the study protocol for the Oregon resident DENV immune cohort. IRBs of the University of Michigan and the Universidad San Francisco de Quito approved the study protocol for the EcoDess Study. The NIDDL and NMRC worked with deidentified clinical samples and a serum panel under approved human use protocol #: PJT-17-06 from NAMRU-2 and NAMRU-6. IRBs of the Hospital Pedro Vicente Maldonado and the Universidad San Francisco de Quito approved the study protocol for the ECUAVIDA Study. The Nicaragua Pediatric Dengue Cohort Study was reviewed and approved by the institutional review boards of the University of California, Berkeley (protocol: 2010-09-2245), the University of Michigan (study ID: HUM00091606), and the Nicaraguan Ministry of Health (protocol NIC-MINSA/CNDR CIRE-09/03/07-008). The Pediatric Dengue Hospital-based Study was reviewed and approved by the institutional review boards of the University of California, Berkeley (protocol 2010-06-1649) and the Nicaraguan Ministry of Health (protocol NIC-MINSA/CNDR CIRE-01/10/06-13). Written informed consent to participate in this study was provided by the participants’ legal guardian/next of kin.

## Author Contributions

LK, FE, JC, EH, and PL conceived the project. PL, AH, IL, ACh, and NM developed the Excivion dengue and Zika RDTs. SH, EH, FC, MM, VS, H-WC, S-JW, MS, AL, and PC performed evaluation of the Excivion RDTs. SM, LK, SV, GT, JE, WM, JC, and ACo evaluated the SD BIOLINE RDT. FE and LK analyzed the data and wrote the manuscript. All authors contributed to the article and approved the submitted version.

## Funding

This work was supported by the Intramural Research Program of the National Institute of Allergy and Infectious Diseases (FE, LK), and National Institutes of Health (NIH) grants (R01AI132372-02, to JE, JC), R21AI135537-02 (to WM), P01AI106695 (to EH), and contracts ‘972224’ and ‘971509’ from the UK Government *via* Innovate UK to Excivion Ltd under the “New Vaccines for Global Epidemics: Development and Manufacture” competition. LK was supported in part by the Global Health Equity Scholar FIC/NIH training grant D43TW010540. WM was also supported by the National Center for Advancing Translational Science CTSA UL1 TR000128, Oregon Clinical and Translational Research Institute, Takeda Vaccines IISR 2016-101586, and the Sunlin and Priscilla Chou Foundation.

## Conflict of Interest

Authors PL and AH were employed by company Excivion Ltd. PL and AH hold stock in Excivion Ltd and via Excivion Ltd hold stock in Coronex Ltd, which companies own patent applications in RDTs and vaccines. ACh was employed by company Oxford Expression Technologies Ltd. NM was employed by company GlobalDX Ltd. IL has acted as a consultant for Excivion Ltd.

The remaining authors declare that the research was conducted in the absence of any commercial or financial relationships that could be construed as a potential conflict of interest.

## Publisher’s Note

All claims expressed in this article are solely those of the authors and do not necessarily represent those of their affiliated organizations, or those of the publisher, the editors and the reviewers. Any product that may be evaluated in this article, or claim that may be made by its manufacturer, is not guaranteed or endorsed by the publisher.

## Supplementary Material

Supplementary Figure 1 Evaluation of the IgG component of the SD BIOLINE Dengue IgG/IgM rapid diagnostic test, stratified by primary DENV infecting serotype, for samples from the Oregon traveler cohort. Columns indicate infecting serotype, top row shows visual evaluations, and bottom row shows quantitative readings.

Supplementary Figure 2 Positive predictive value and negative predictive value estimates for the EcoDess, Nicaragua, and ECUAVIDA cohorts. Positive predictive values and negative predictive values using optimal ≥98% specificity (PPV: light orange dots, NPV: blue squares) and minimum ≥90% specificity (PPV: dark orange dots, NPV: navy blue squares) at distinct timepoints for the EcoDess **(A),** Nicaragua (using seroprevalence estimates from both 2004 and 2015) **(B),** and ECUAVIDA **(C)** cohorts. Smoothing splines and simple linear regressions show trends over time for the EcoDess and Nicaragua cohorts respectively.

Supplementary Figure 3 Evaluation of the Excivion Dengue IgG rapid diagnostic test by primary and secondary DENV infecting serotype in samples from the Nicaragua Pediatric Dengue Cohort and Hospital-based Studies. Columns indicate infecting serotype, top row shows visual evaluations, and bottom row shows quantitative readings.

Supplementary Table 1 Sensitivity and specificity values at each timepoint for the Oregon Traveler Cohort evaluated using the SD BIOLINE Dengue IgG/IgM RDT (estimate and 95% confidence intervals are shown).

Supplementary Table 2 Sensitivity and specificity values at each timepoint for the EcoDess Cohort evaluated using the SD BIOLINE Dengue IgG/IgM RDT (estimate and 95% confidence intervals are shown).

Supplementary Table 3 Sensitivity and specificity values at each timepoint for the Nicaragua Cohort samples evaluated using the Excivion Dengue RDT (estimate and 95% confidence intervals are shown).

Supplementary Table 4 Sensitivity and specificity values at each timepoint for the Nicaragua Cohort samples evaluated using the Excivion Zika RDT (estimates and 95% confidence intervals are shown).
