# Supplementary figures and images for: Adapting Rapid Diagnostic Tests to Detect Historical Dengue Virus Infections
Source: Front Immunol. 2021 Jul 23;12:703887. doi: 10.3389/fimmu.2021.703887 (PMC8344047; doi:10.3389/fimmu.2021.703887)

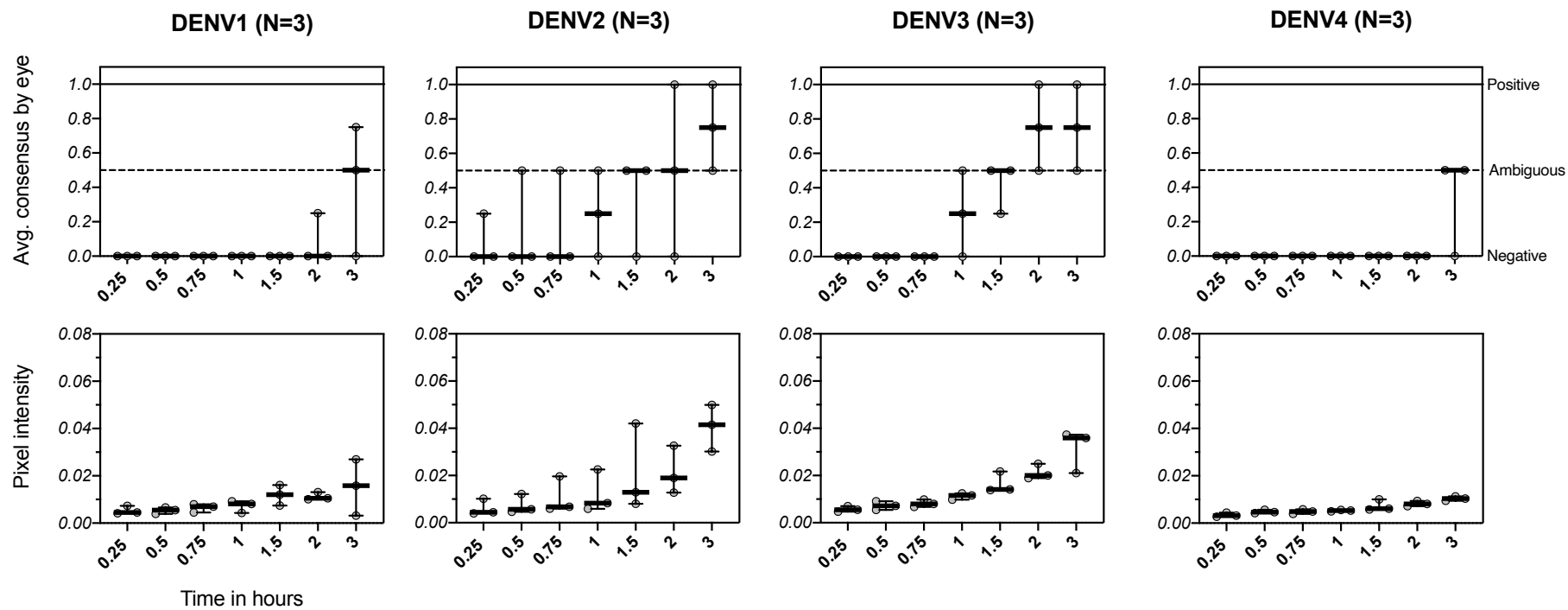

Fig. S1

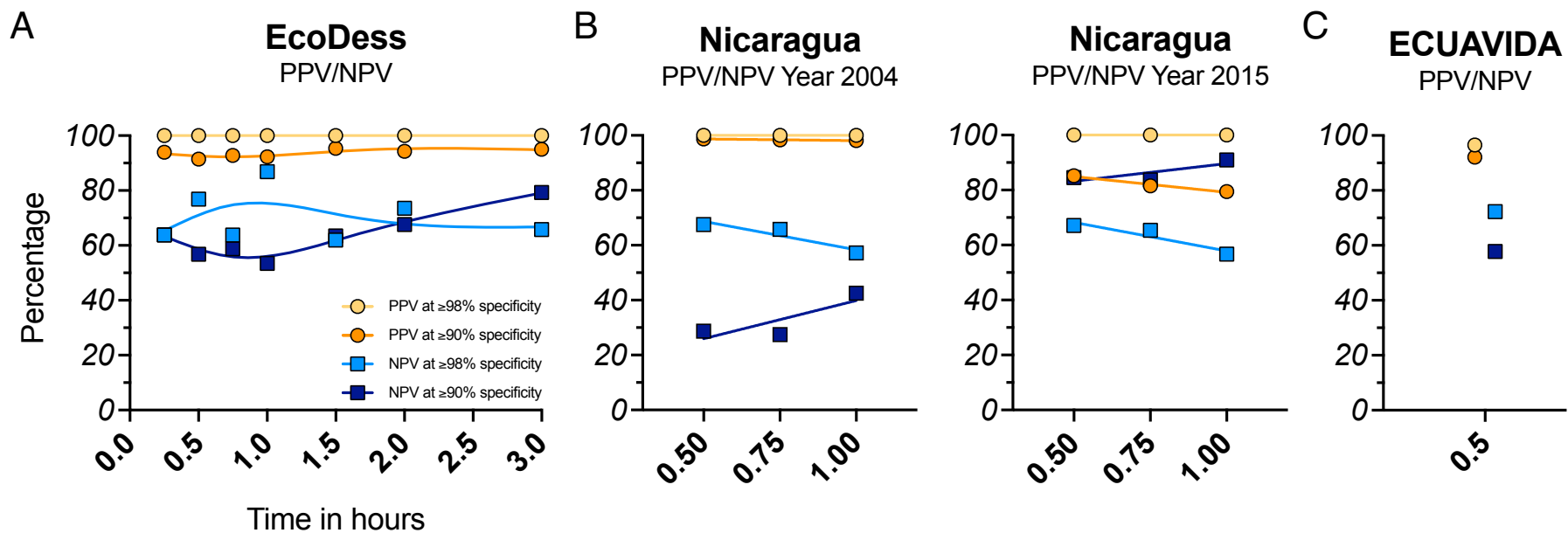

Fig. S2

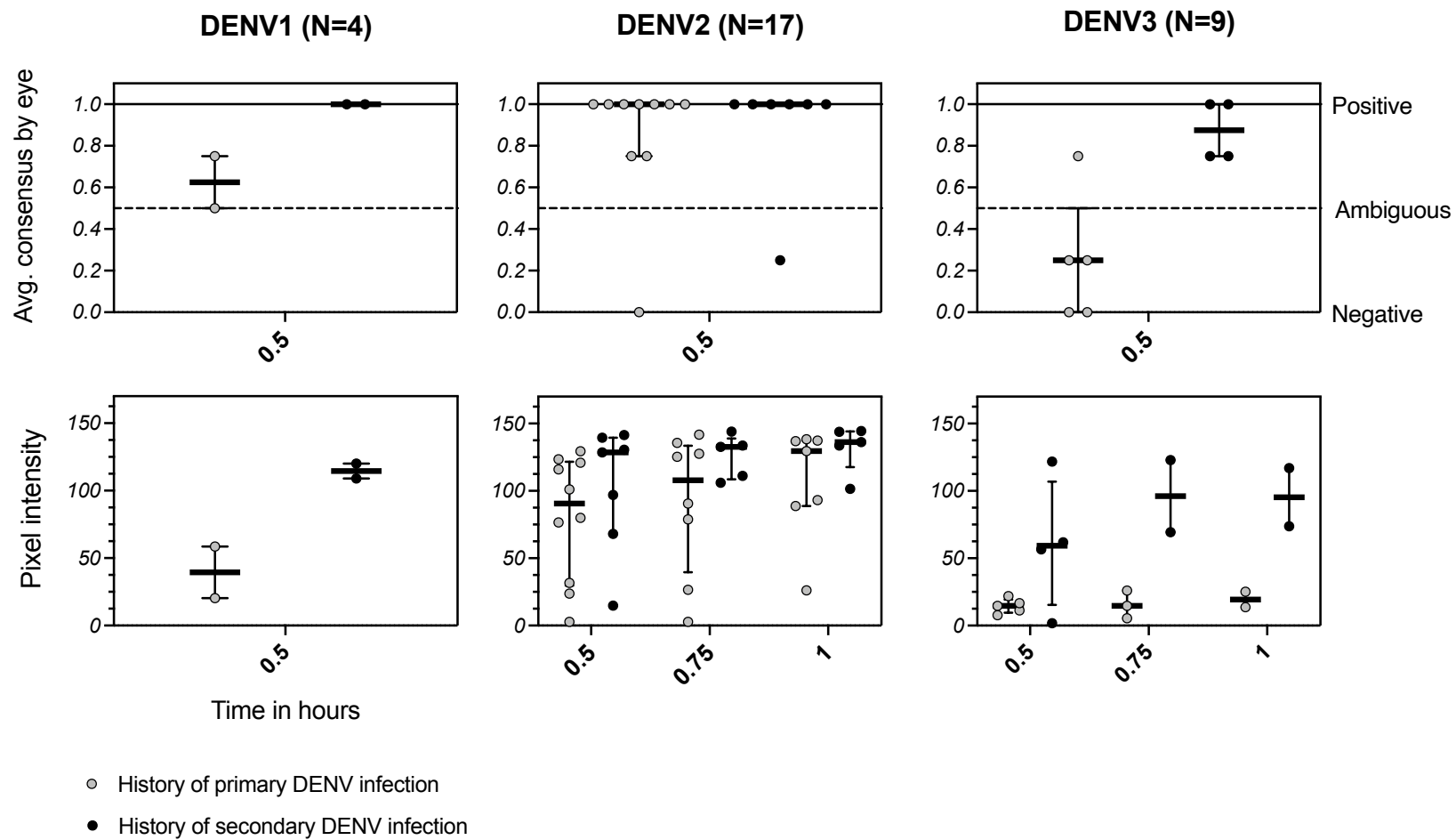

Fig. S3

Supplement: Supplementary Figure 1 — Evaluation of the IgG component of the SD BIOLINE Dengue IgG/IgM rapid diagnostic test, stratified by primary DENV infecting serotype, for samples from the Oregon traveler cohort. Columns indicate infecting serotype, top row shows visual evaluations, and bottom row shows quantitative readings. [file Presentation_1.pdf]
