# Supplementary material for: Adapting Rapid Diagnostic Tests to Detect Historical Dengue Virus Infections
Source: Front Immunol. 2021 Jul 23;12:703887. doi: 10.3389/fimmu.2021.703887 (PMC8344047; doi:10.3389/fimmu.2021.703887)
Supplement: Supplementary file 2 [file DataSheet_1.pdf]

Table S1. Sensitivity and specificity values at each timepoint for the Oregon Traveler Cohort evaluated using the SD BIOLINE Dengue IgG/IgM RDT (estimate and 95% confidence intervals are shown).

**Timepoint**	**0.25 hrs**	**0.5 hrs**	**0.75 hrs**	**1 hr**	**1.5 hrs**	**2 hrs**	**3 hrs**
**All DENV+ samples**							
Qualitative Sensitivity	0 (0-19)	0 (0-19)	0 (0-19)	6 (0-28)	13 (2-36)	38 (18-61)	53 (30-75)
Qualitative Specificity	100 (68-100)	100 (68-100)	100 (68-100)	100 (68-100)	100 (68-100)	100 (68-100)	100 (68-100)
Quantitative Sensitivity at Optimal (≥98%) and Minimum (≥90%) Specificity	44 (23-67)	88 (64-98)	81 (57-93)	25 (10-50)	63 (39-82)	56 (33-77)	67 (70-100)
Optimal (≥98%) and Minimum (≥90%) Quantitative Specificity	100 (68-100)	100 (68-100)	100 (68-100)	100 (68-100)	100 (68-100)	100 (68-100)	100 (68-100)
**Only DENV+ primary samples**							
Qualitative Sensitivity	0 (0-24)	0 (0-24)	0 (0-24)	0 (0-24)	0 (0-24)	25 (9-53)	42 (19-68)
Qualitative Specificity	100 (68-100)	100 (68-100)	100 (68-100)	100 (68-100)	100 (68-100)	100 (68-100)	100 (68-100)

Table S2. Sensitivity and specificity values at each timepoint EcoDess Cohort, SD BIOLINE Dengue IgG/IgM RDT (estimate and 95% confidence intervals).

**Timepoint**	**0.25 hrs**	**0.5 hrs**	**0.75 hrs**	**1 hr**	**1.5 hrs**	**2 hrs**	**3 hrs**
**All DENV+ samples**							
Qualitative Sensitivity	41 (28-56)	70 (53-82)	80 (66-89)	96 (85-99)	97 (87-100)	95 (85-99)	98 (88-100)
Qualitative Specificity	100 (87-100)	100 (84-100)	96 (79-100)	80 (61-91)	88 (66-98)	76 (57-89)	36 (20-55)
Quantitative Sensitivity at Optimal (≥98%) Specificity	57 (42-70)	31 (64-98)	57 (42-70)	16 (10-50)	62 (46-75)	36 (24-51)	52 (38-66)
Optimal (≥98%) Quantitative Specificity	100 (87-100)	100 (84-100)	100 (86-100)	100 (87-100)	100 (82-100)	100 (87-100)	100 (87-100)
Quantitative Sensitivity at Minimum (≥90%) Specificity	70 (56-82)	61 (45-75)	64 (49-76)	55 (40-68)	69 (54-81)	75 (61-85)	86 (73-94)
Minimum (≥90%) Quantitative Specificity	92 (75-99)	90 (70-98)	91 (73-98)	92 (75-99)	94 (73-100)	92 (75-99)	92 (75-99)
**Only DENV+ primary samples**							
Qualitative Sensitivity	16 (6-38)	44 (23-67)	53 (32-73)	79 (57-91)	83 (61-94)	95 (75-100)	95 (75-100)
Qualitative Specificity	100 (87-100)	100 (84-100)	100 (86-100)	92 (75-99)	88 (66-98)	80 (61-91)	40 (23-59)

Table S3. Sensitivity and specificity values at each timepoint for the Nicaragua Cohort samples evaluated using the Excivion Dengue RDT (estimate and 95% confidence intervals are shown).

**Timepoint**	**0.5 hrs**	**0.75 hrs**	**1 hr**
**All DENV+ samples**			
Qualitative Sensitivity	79 (62-89)	Not collected	Not collected
Qualitative Specificity	79 (57-92)	Not collected	Not collected
Quantitative Sensitivity at Optimal (≥98%) Specificity	49 (33-65)	52 (32-72)	75 (51-90)
Optimal (≥98%) Quantitative Specificity	100 (83-100)	100 (80-100)	100 (72-100)
Quantitative Sensitivity at Minimum (≥90%) Specificity	64 (47-78)	62 (41-79)	81 (57-93)
Minimum (≥90%) Quantitative Specificity	95 (75-100)	93 (70-100)	90 (60-100)
**Only DENV+ primary samples**			
Qualitative Sensitivity	67 (44-84)	Not collected	Not collected
Qualitative Specificity	79 (57-91)	Not collected	Not collected

Table S4. Sensitivity and specificity values at each timepoint for the Nicaragua Cohort samples evaluated using the Excivion Zika RDT (estimates and 95% confidence intervals are shown).

**Timepoint**	**0.5 hrs**	**0.75 hrs**	**1 hr**
Qualitative Sensitivity	100 (89-100)	Not collected	Not collected
Qualitative Specificity	70 (40-89)	Not collected	Not collected
Quantitative Sensitivity at Optimal (≥98%) Specificity	93 (79-99)	97 (83-100)	100 (89-100)
Optimal (≥98%) Quantitative Specificity	100 (72-100)	100 (72-100)	100 (72-100)
Quantitative Sensitivity at Minimum (≥90%) Specificity	100 (89-100)	100 (89-100)	100 (89-100)
Minimum (≥90%) Quantitative Specificity	90 (60-100)	90 (60-100)	90 (60-100)
